# National Adolescent School-based Health Survey - PeNSE 2015: Sedentary behavior and its correlates

**DOI:** 10.1371/journal.pone.0228373

**Published:** 2020-01-30

**Authors:** Roberta Mendes Abreu Silva, Amanda Cristina de Souza Andrade, Waleska Teixeira Caiaffa, Danielle Souto de Medeiros, Vanessa Moraes Bezerra

**Affiliations:** 1 Multidisciplinary Institute in Health, Anísio Teixeira Campus, Federal University of Bahia, Vitória da Conquista, Bahia, Brazil; 2 Federal University of de Mato Grosso, Cuiabá, Mato Grosso, Brazil; 3 Faculty of Medicine, Federal University of Minas Gerais, Belo Horizonte, Minas Gerais, Brazil; 4 Observatory for Urban Health in Belo Horizonte, Belo Horizonte, Minas Gerais, Brazil; University Sains Malaysia, MALAYSIA

## Abstract

**Objective:**

To investigate the association between sedentary behavior (SB) and sociodemographic, social support, behavioral, and health variables among Brazilian adolescents.

**Methods:**

The 2015 National Adolescent School-based Health Survey (PeNSE) was a cross-sectional study consisting of 102,072 Brazilian ninth-graders (mainly aged 13–15 years). SB was defined as the time (in hours) watching television, using a computer, playing video games, talking to friends, or doing other activities in a sitting position. For analysis purposes, SB was categorized into different cut-offs as per the sample distribution quartiles: >2 versus <2 (25^th^ percentile); >4 versus <4 (50^th^ 26 percentile) and >6 versus <6 (75^th^ 27 percentile). We employed Poisson univariate and multivariate regression analyses with robust variance and hierarchical entry of variables for each cut-off point.

**Results:**

The prevalence rates of each SB cut-off point were 68.15% (CI: 67.44–68.86), 44.15% (CI: 43.40–44.90) and 24.97% (CI:24.37–25.57) for >2, >4 and >6 hours, respectively. The following characteristics were positively and significantly associated with each SB cut-off point in the final models: females, current employment, higher household economic status and higher maternal schooling, lower levels of parents checking homework, tobacco and alcohol use, soft drink and fruit consumption, and regular, poor or very poor self-assessed health status. Conversely, students who self-declared brown were less likely to be classified as a SB cut-off point. Significant associations with age, report of close friends, and physical activity varied by different SB cut-off points.

**Conclusion:**

Understanding the SB correlates in their different dimensions contributes to the identification of subgroups of adolescents with higher SB prevalence, which is crucial in the development and improvement of public policies. The demographic and behavioral characterization of these groups can guide the development of future intervention strategies, considering the school and family contexts of these adolescents.

## Introduction

There is an ongoing debate in the scientific community about sedentary behavior (SB), its consequences for health, and its impact on different stages of life [1, 2]. SB has been associated with the occurrence of adverse physical and mental health outcomes, such as diabetes, cancer, metabolic syndrome, obesity, cardiovascular disorders, mortality [2–5], anxiety, self-esteem, and depression [6, 7]. That is, the risk of developing such diseases increases with increasing SB time.

SB is defined by activities performed while in a sitting, reclining, or lying posture, with energy cost equal to or less than 1.5 metabolic equivalents (MET). Activities in this position include the use of electronic devices, reading, writing, drawing, painting, doing homework, sitting at school, on the bus, car, or train [8].

It is essential to understand the factors associated with the SB among adolescents, considering the profound changes related to psychological, social, and health risk behaviors at this stage of life that, once started, may continue into adulthood [2].

An increasing number of studies are investigating SB-associated factors in Brazilian adolescents, showing associations with age, region of residence, school shift, occupational status, consumption of high energy content foods, and soft drinks [2,9]. However, a recent systematic literature review found that most of the studies define sedentary behavior as self-reported screen time only [9]. Therefore, some topic-related gaps must still be filled. The main gaps are the need for SB investigation other than screen time and the definitions of SB cutoffs for the adolescent population [10–12].

This study aims to investigate the association of sociodemographic, social support, behavioral, and health domains with sedentary behavior, considering different cut-off points among Brazilian adolescents.

## Methods

This study analyzed data from the Third edition of the National Adolescent School-based Health Survey, conducted in 2015 (PeNSE, in Portuguese), and previous editions were held in 2009 and 2012. This is a cross-sectional study representative of Brazil, among ninth-graders enrolled at and attending public and private schools. PeNSE data is available on the Brazilian Institute of Geography and Statistics (IBGE) website. The survey was approved by the National Research Ethics Commission (CONEP) and registered under protocol number 1.006.467.

Fifty-three geographic strata were defined, including the 26 state capitals, the remaining municipalities within each state, and the Federal District to select the nationwide sample. The sample size was calculated to provide estimates in each stratum, considering an estimated proportion of 50% with a maximum sample estimate variance. The expected sample of 128,027 students was designed to have a 3% margin of error and a 95% confidence level.

A sample of schools was defined using systematic sampling with a probability proportional to the number of schools in the strata (n = 3,160) in each stratum. Classes were randomly selected. One class was selected in the schools with two or three classes, and two classes were selected in the schools with more than three classes, totaling 4,159 classes. All the adolescents in the selected classes were eligible to participate in the survey and were invited to complete the questionnaire. A total of 102,072 individuals participated in the survey.

Data was collected during class hours using smartphones made available by the IBGE. An application on each device contained a self-administered and structured questionnaire divided into thematic modules. Students could answer the questionnaire fully or partially. Data was transmitted and processed daily, allowing smartphones and the database to be updated automatically. See Oliveira et al. [13] for further details on sample selection and data collection.

### Variables description

**Fig 1:**
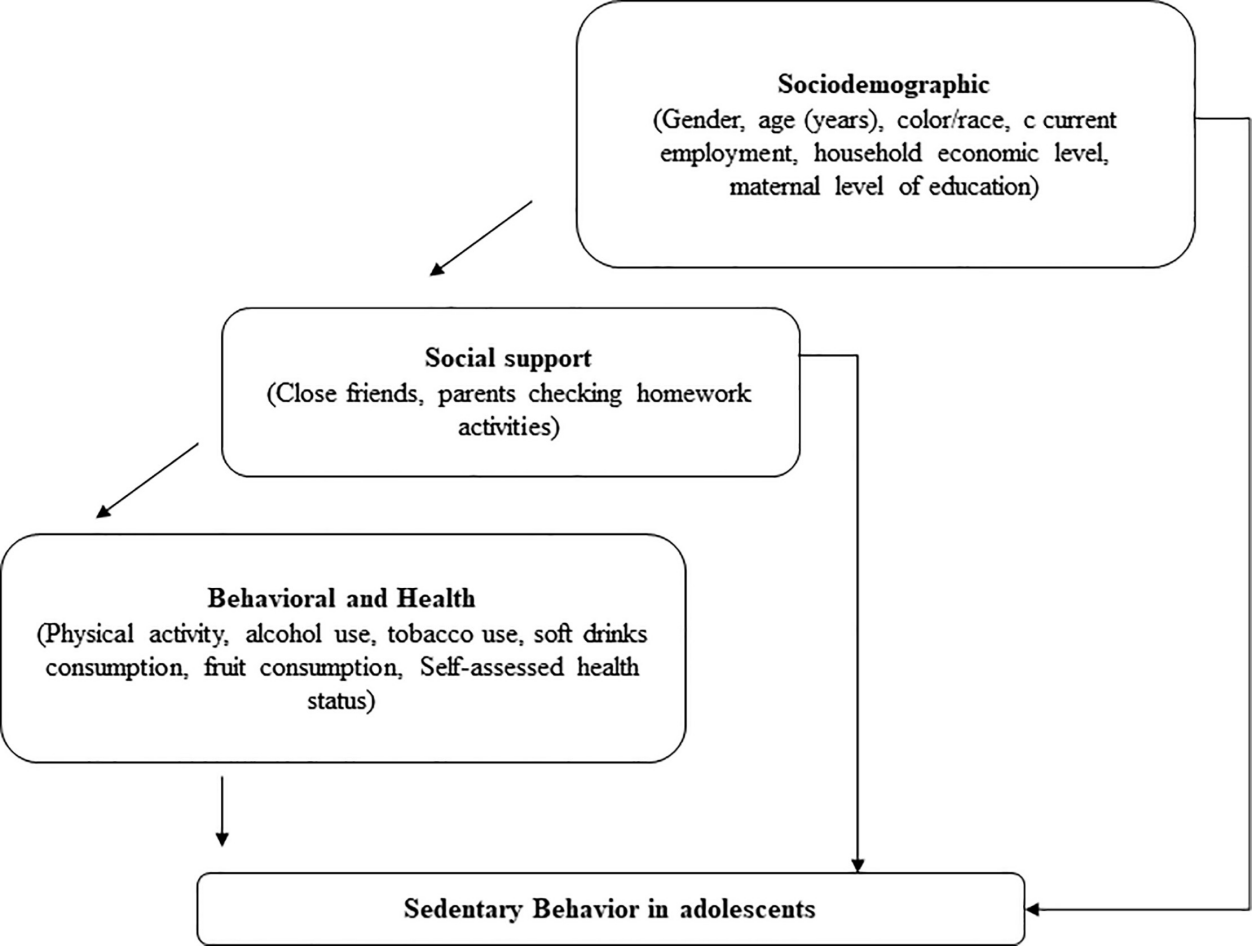
Conceptual model on sedentary behavior for adolescents.

Sedentary behavior (SB) was defined in the questionnaire as follows: “On a typical weekday, how much time do you spend sitting, watching television, using a computer, playing video games, talking to friends, or doing other activities while sitting? (Excluding Saturday, Sunday, holidays and time at school)”. This question was adapted from a similar question on the Global School-based Student Health Survey [14]. For analysis purposes, SB was categorized into different cut-offs as per the sample distribution quartiles: >2 versus <2 (25th percentile); >4 versus <4 (50th 26 percentile) and >6 versus <6 (75th 27 percentile). Independent variables were selected from a conceptual model on SB and structured in three blocks (Fig 1).

The sociodemographic block included factors such as gender, age, self-identified skin color/race (white, black, brown, yellow, and indigenous) [15], employment status, maternal schooling, and household economic status score. The score was calculated based on the ownership of the following goods and services: ownership of a landline, cell phone, computer, internet, automobile, motorcycle, indoor bathroom, and presence of housekeeper three times a week or more. Each item received a score corresponding to the inverse number or presence of the item in a given household. Scores from all items were added up to obtain the final score, and analyzed in tertial distribution, where the lowest third were households with lower access to goods and services [16]. The social support block included two variables: the existence of close friends and parents checking homework activities.

The behavioral and health block consisted of the following variables: fruit consumption (≥5 days a week), soft drink consumption (≥5 days a week) [17], alcohol use (used in the last 30 days), and regular tobacco use (used in the last 30 days). Physical activity was also considered, calculated as the mean daily time employed by teenagers to perform the following physical activities in the seven days before the survey: commuting to school, physical education classes, and other extracurricular physical activities. Those who performed 300 minutes or more of weekly exercise were considered active [18]. Self-assessed health status was evaluated by the question “How would you rate your health status?” and categorized as good/very good, regular, and poor/very poor.

### Missing data

From the total number of PeNSE 2015 respondents, 25% (n = 25,434) did not report their mother’s schooling level. We used multiple imputations by chained equations to assign numerical values to missing observations. Gender, housekeepers, and home internet access were used as predictive variables based on previous literature [19].

### Data analysis

A univariate analysis between SB and independent variables was conducted using Poisson regression with a robust variance to compare prevalence ratios (PR) at a 5% confidence level. Concerning multivariate analysis, we introduced variables from thematic blocks into the model in a hierarchical fashion, as follows: 1) sociodemographic, 2) social support, and 3) behavioral and health variables. Variables in the most distal blocks served as adjustment factors for hierarchically inferior blocks. The Akaike information criterion was adopted to compare the three models. The multivariate models included all variables with p ≤ 0.20 in the univariate model. All analyses were performed using Stata version 12.0.

## Results

Most adolescents in the sample (n = 102,072) were female (51.3%), older than 14 years (51.0%), were not currently employed (86.6%), and identified as brown or black (56.5%). Less than half (32.4%) of adolescents were from households with lower economic standing, while 51.6% had mothers who did not complete high school. In the social support block, 76.9% reported having three or more friends, and 44.4% said that their parents never or rarely checked their homework. We observed that 34.4% of students were classified as physically active, and 76.7% and 94.4% did not use alcohol and tobacco, respectively. Moreover, 73.3% of students regularly consumed soft drinks, and 67.3% consumed fruit less than five days a week. Most adolescents (73.0%) reported good or very good self-assessed health (Table 1).

**Table 1:** Description of the sample according to sociodemographic, social support, behavioral and health variables—PeNSE, 2015.

Variables	*%	^†^95% CI
**Block 1: Sociodemographic**		
**Gender**		
Male	48.7	48.1–49.3
Female	51.3	50.7–51.9
**Age (years)**		
≥ 16	11.0	10.4–11.5
15	19.8	19.1–20.5
14	51.0	50.1–51.9
≤13	18.2	17.2–19.3
**Color/Race**		
White	36.1	35.1–37.2
Black	13.4	12.9–13.9
Yellow	4.1	3.9–4.4
Brown	43.1	42.2–43.9
Indigenous	3.3	3.1–3.5
**Household economic level**		
High	31.0	29.8–32.1
Medium	36.6	35.8–37.4
Lower	32.4	31.4–33.4
**Maternal level of education**		
No study	7.7	7.3–8.1
Incomplete primary school	26.7	25.9–27.4
Primary school/incomplete high school	17.2	16.6–17.8
High school/ incomplete higher education	30.6	29.9–31.3
Higher Education	17.8	16.8–18.7
**Current employment**		
Yes	13.4	12.9–13.9
No	86.6	86.1–87.1
**Block 2: Social support**		
**Close friends**		
No friend	4.3	4.0–4.5
1 or 2 friends	18.8	18.3–19.4
3 or more	76.9	76.3–77.4
**Parents checking homework activities**		
Most of the times / always	31.9	31.2–32.5
Sometimes	23.8	23.3–24.2
Never or rarely	44.4	43.7–45.1
**Block 3: Behavioral and Health**		
**Physical activity**		
Inactive	65.6	64.9–66.4
Active	34.4	33.6–35.1
**Alcohol use**		
No	76.7	76.1–77.3
Yes	23.3	22.7–23.9
**Tobacco use**		
No	94.4	94.1–94.8
Yes	5.6	5.2–5.9
**Soft drinks consumption**		
< 5 day/week	73.3	72.7–74.0
≥ 5 day/week	26.7	26.0–27.3
**Fruit consumption**		
≥ 5 day/week	32.7	32.1–33.4
< 5 day/week	67.3	66.6–67.9
**Self-assessed health status**		
Very Good / good	73.0	72.5–73.6
Regular	19.9	19.4–20.4
Poor/very poor	7.1	6.8–7.4

*Percentage (%) Frequency

†95% CI: 95% Confidence Interval.

In this sample, 68.1% (95% CI: 67.4–68.9) of adolescents reported >2 hours of SB; 44.1% (95% CI: 43.4–44.9) and 25.0% (95% CI:24.4–25.6) reported >4 and >6 hours, respectively.

In the univariate analysis, all three SB cut-off points were positively associated with females, currently working, regular alcohol use, regular tobacco use, and soft drink consumption. A dose-response effect was observed in the association with age, economic level, maternal education level, parents checked homework activities, and self-assessed health status. White adolescents were more likely than black and brown adolescents to spend time reporting SB at all three cut-off points and more likely than indigenous adolescents to be sedentary at the >2 and >4 hours range. SB was positively associated with the existence of close friends and physical activity at the >2 hours, while regular fruit consumption was significant at the >2 and >4 hours range (Table 2).

**Table 2:** Prevalence of sedentary behavior prevalence according to sociodemographic, social support, behavioral, and health variables—PeNSE, 2015.

Variables	SB > 2 hours			SB > 4 hours			SB >6 hours		
	*P%	^†^PR	^‡^ 95% CI	*P%	^†^PR	^‡^ 95% CI	*P%	^†^PR	^‡^ 95% CI
**Block 1: Sociodemographic**									
**Gender**									
Male	67.3	1.00	-	42.3	1.00	-	22.5	1.00	-
Female	69.0	1.02	1.01–1.04	45.9	1.08	1.05–1.11	27.3	1.22	1.17–1.27
**Age (years)**									
≥ 16	56.9	1.00	-	36.8	1.00	-	22.0	1.00	-
15	63.8	1.12	1.09–1.16	41.6	1.13	1.07–1.19	24.1	1.10	1.02–1.18
14	70.7	1.24	1.21–1.28	46.0	1.25	1.20–1.30	25.7	1.17	1.10–1.24
≤13	72.5	1.28	1.24–1.32	46.2	1.25	1.19–1.32	27.8	1.17	1.09–1.26
**Color/Race**									
White	72.4	1.00	-	47.0	1.00	-	26.3	1.00	-
Black	63.3	0.88	0.85–0.90	41.0	0.87	0.83–0.92	23.9	0.91	0.85–0.97
Yellow	71.0	0.98	0.95–1.02	47.9	1.02	0.96–1.08	28.1	1.07	0.98–1.16
Brown	66.1	0.91	0.90–0.93	42.5	0.90	0.88–0.93	23.8	0.90	0.86–0.95
Indigenous	65.3	0.90	0.86–0.94	43.6	0.93	0.86–0.99	26.0	0.98	0.89–1.10
**Household economic level**									
High	75.8	1.00	-	50.8	1.00	-	28.7	1.00	-
Medium	71.8	0.94	0.93–0.96	47.5	0.93	0.91–0.96	27.0	0.94	0.90–0.98
Lower	56.9	0.75	0.73–0.77	34.2	0.67	0.65–0.70	19.2	0.67	0.63–0.70
**Maternal level of education**									
No study	51.4	1.00	-	32.0	1.00	-	19.1	1.00	-
Incomplete primary school	62.4	1.21	1.17–1.27	39.5	1.23	1.16–1.31	22.2	1.17	1.06–1.28
Primary school/incomplete high school	68.8	1.34	1.28–1.40	45.1	1.41	1.31–1.51	25.6	1.34	1.22–1.48
High school/ incomplete higher education	73.3	1.42	1.36–1.49	48.1	1.50	1.41–1.60	27.0	1.42	1.29–1.55
Higher Education	74.7	1.45	1.39–1.52	48.7	1.52	1.41–1.63	27.6	1.45	1.31–1.60
**Current employment**									
Yes	63.9	1.00	-	40.2	1.00	-	22.6	1.00	-
No	68.8	1.08	1.05–1.10	44.8	1.11	1.07–1.16	25.3	1.12	1.05–1.19
**Block 2: Social support**									
**Close friends**									
No friend	64.5	1.00	-	43.3	1.00	-	25.2	1.00	-
1 or 2 friends	68.2	1.06	1.01–1.10	44.8	1.03	0.96–1.11	25.9	1.03	0.92–1.15
3 or more	68.4	1.06	1.02–1.10	44.1	1.02	0.95–1.09	24.8	0.98	0.89–1.09
**Parents checking homework activities**									
Most of the times / always	61.4	1.00	-	36.3	1.00	-	20.0	1.00	-
Sometimes	68.2	1.11	1.09–1.13	42.4	1.17	1.12–1.21	21.9	1.10	1.04–1.16
Never or rarely	73.0	1.19	1.17–1.21	50.8	1.40	1.34–1.44	30.2	1.51	1.45–1.58
**Block 3: Behavioral and Health**									
**Physical activity**									
Inactive	67.3	1.00	-	44.4	1.00	-	25.8	1.00	-
Active	69.8	1.04	1.02–1.05	43.6	0.98	0.96–1.01	23.5	0.91	0.88–0.95
**Alcohol use**									
No	66.1	1.00	-	41.7	1.00	-	22.8	1.00	-
Yes	75.1	1.14	1.12–1.16	52.2	1.25	1.22–1.28	32.2	1.41	1.36–1.47
**Tobacco use**									
No	67.8	1.00	-	43.6	1.00	-	24.5	1.00	-
Yes	74.3	1.10	1.07–1.13	52.7	1.21	1.15–1.27	33.2	1.35	1.24–1.48
**Soft drinks consumption**									
< 5 day/week	65.3	1.00	-	40.2	1.00	-	21.4	1.00	-
≥ 5 day/week	76.1	1.17	1.15–1.18	55.1	1.37	1.34–1.41	34.9	1.63	1.57–1.70
**Fruit consumption**									
≥ 5 day/week	66.9	1.00	-	42.8	1.00	-	24.2	1.00	-
< 5 day/week	68.8	1.03	1.01–1.05	44.9	1.05	1.02–1.08	25.3	1.05	0.99–1.09
**Self-assessed health status**									
Very Good / good	66.9	1.00	-	42.1	1.00	-	23.1	1.00	-
Regular	72.1	1.08	1.06–1.10	49.6	1.18	1.14–1.21	29.7	1.28	1.23–1.35
Poor/very poor	71.2	1.06	1.03–1.09	51.0	1.21	1.16–1.26	31.6	1.37	1.29–1.45

*P: prevalence of sedentary behavior

^†^PR: prevalence ratio

^‡^CI: 95% Confidence Interval

Similar results were observed in the multivariate analysis. For all three cut-off points, SB was associated with gender, brown skin color/race, economic level, current employment, maternal education level, parents checking homework activities, alcohol and tobacco use, fruit and soft drink consumption, and self-assessed health status.

Age was only significant at >2 and >4 hours SB. Regarding skin color/race, indigenous and black were significant only at >2 hours’ cut-off points compared to whites. Also, reporting three or more close friends and physically active students was significantly associated with SB at >2 hours’ cut-off points. The estimated point prevalence ratios in the multivariate models decreased for all SB cut-off points (Table 3).

**Table 3:** Multivariate analysis between sedentary behavior and sociodemographic, social support, behavioral and health variables—PeNSE– 2015.

Variables	SB > 2 hours		SB > 4 hours		SB >6 hours	
	^†^PR	^‡^95% CI	^†^PR	^‡^95% CI	^†^PR	^‡^95% CI
Block 1: Sociodemographic*						
**Gender**						
Male	1.00	-	1.00	-	1.00	-
Female	1.02	1.01–1.04	1.09	1.06–1.12	1.24	1.19–1.29
**Age (years)**						
≥ 16	1.00	-	1.00	-		
15	1.08	1.04–1.11	1.07	1.02–1.12		
14	1.14	1.11–1.17	1.11	1.06–1.16		
≤13	1.15	1.11–1.18	1.08	1.02–1.14		
**Color/Race**						
White	1.00	-	1.00	-	1.00	-
Black	0.94	0.91–0.97	0.95	0.91–1.01	0.99	0.93–1.07
Yellow	1.01	0.97–1.04	1.05	0.99–1.11	1.09	0.99–1.18
Brown	0.96	0.94–0.97	0.96	0.93–0.99	0.96	0.91–0.99
Indigenous	0.96	0.92–0.99	0.99	0.93–1.07	1.06	0.95–1.19
**Household economic level**						
High	1.00	-	1.00	-	1.00	-
Medium	0.97	0.95–0.98	0.95	0.92–0.98	0.94	0.90–0.99
Lower	0.80	0.79–0.82	0.71	0.69–0.74	0.69	0.65–0.73
**Maternal level of education**						
No study	1.00	-	1.00	-	1.00	-
Incomplete primary school	1.15	1.11–1.20	1.16	1.09–1.23	1.10	1.01–1.21
Primary school/incomplete high school	1.23	1.18–1.29	1.27	1.19–1.37	1.23	1.11–1.36
High school/ incomplete higher education	1.28	1.22–1.33	1.31	1.22–1.39	1.25	1.14–1.37
Higher Education	1.25	1.19–1.30	1.25	1.16–1.35	1.21	1.09–1.34
**Current employment**						
Yes	1.00	-	1.00	-	1.00	-
No	1.06	1.04–1.09	1.10	1.06–1.14	1.09	1.03–1.16
Block 2: Social support**						
**Close friends**						
No friend	1.00	-				
1 or 2 friends	1.04	1.00–1.08				
3 or more	1.04	1.01–1.09				
**Parents checking homework activities**						
Most of the times / always	1.00	-	1.00	-	1.00	-
Sometimes	1.10	1.08–1.12	1.16	1.11–1.20	1.16	1.12–1.20
Never or rarely	1.18	1.16–1.20	1.38	1.34–1.42	1.38	1.34–1.42
Block 3: Behavioral and Health***						
**Physical activity**						
Inactive	1.00	-				
Active	1.04	1.02–1.05				
**Alcohol use**						
No	1.00	-	1.00	-	1.00	-
Yes	1.11	1.09–1.13	1.16	1.13–1.19	1.15	1.12–1.18
**Tobacco use**						
No	1.00	-	1.00	-	1.00	-
Yes	1.05	1.02–1.09	1.09	1.03–1.15	1.07	1.02–1.13
**Soft drinks consumption**						
< 5 day/week	1.00	-	1.00	-	1.00	-
≥ 5 day/week	1.13	1.11–1.14	1.30	1.27–1.34	1.30	1.26–1.33
**Fruit consumption**						
≥ 5 day/week	1.00	-	1.00	-	1.00	-
< 5 day/week	1.03	1.02–1.05	1.03	1.01–1.06	1.03	1.01–1.06
**Self-assessed health status**						
Very Good / good	1.00	-	1.00	-	1.00	-
Regular	1.07	1.05–1.09	1.14	1.11–1.17	1.14	1.12–1.21
Poor/very poor	1.06	1.03–1.09	1.17	1.13–1.22	1.17	1.12–1.21

*Adjusted by block 1 variables.

**Adjusted by block 1 & 2 variables.

*** Adjusted by block 1, 2 & 3 variables.

^†^PR: prevalence ratio

^‡^ CI: 95% Confidence Interval

## Discussion

This study showed that SB in Brazilian adolescents, assessed using different cut-off points, was associated with sociodemographic characteristics (gender, age, color/race, household economic status, student’s employment, and maternal education level), social support (existence of close friends and parents who regularly checked homework activities), and behavioral and health factors (physical activity, alcohol use, tobacco use, fruit consumption, and soft drink consumption and self-assessed health status).

Girls had a higher SB prevalence than boys at all cut-off points, as reported in previous studies [3, 20]. Time spent on the telephone, listening to music, doing homework, writing, talking, and parent restrictions on girls’ participation in outdoor activities may contribute to the gender disparity in SB [21, 22].

Regarding age, our results indicate that younger students spent more time engaging in SB, in line with other studies [23]. This could be explained by the higher amount of free time available to young people [3, 23, 24], and because young people are under surveillance with more parental rules, restricting their activities to the domestic space and, consequently, increasing time spent using electronic devices, reading, and engaging in other sedentary activities [25].

The self-reported black and indigenous color/race was negatively associated with the cut-off point of more than two hours of SB, and brown for all the cut-off points in our sample population. Studies have shown conflicting results regarding the association of SB with color/race among adolescent [26, 27]. Self-assessed color/race should not be considered in the biological sense only, but a surrogate of social, environmental, and economic inequities. [28]. In this study, the discrepancies observed between racial groups and the SB may reflect different socioeconomic status also observed in this population. More impoverished adolescents also had lower SB prevalence, an association already observed in the literature [29]. Lower economic levels may reduce households’ access to goods and services, resulting in lower levels of sedentary activities.

Moreover, in the sociodemographic variables’ block, higher maternal education was positively associated with SB among Brazilian adolescents at all assessed cut-off points. Arguments for this association are based on the highest purchasing power and influences of the number of goods (such as television sets and other electronic devices) available to adolescents in their home environment [27]. Also, working mothers who spend fewer hours at home directly supervising their children may lead to increased use of electronic devices by adolescents while they are on their own [28].

In this study, non-working Brazilian adolescents had a higher SB prevalence compared to those working. A possible explanation for this finding is that unemployed adolescents have fewer social commitments after school and, therefore, more free time to engage in sedentary activities [26]. Unemployed adolescents may also experience reduced parental supervision and limitations regarding the use of screen devices [26], and may, therefore, have more opportunities to develop SB after school hours.

According to the findings in the social support block, adolescents who had three or more friends and whose parents never or rarely checked their homework activities had a higher SB prevalence. This finding was only detected in the >2 hours’ cut-off point. Studies with similar findings suggest that habits and behaviors developed by friends influence and reinforce adolescents’ behavior since adolescents are prone to reproduce the behavior of people close to them [29, 30]. Similarly, other studies highlight the special relevance of family interactions and the social environment for this particular population sub-group [31, 32].

Physically active adolescents also evidenced a higher SB prevalence at the > 2 hours’ cut-off point. The relationship between SB and physical activity is not yet consistent in the literature [23, 24, 33, 34]. Our results differ from previous studies that report a negative association between physical activity and SB [23, 24], especially with moderate to vigorous physical activity [35]. However, SB is a distinct construct of physical activity, with its form of measurement and determinants. It is not merely characterized by the absence of physical activity but by a set of activities performed in the sitting position, which represent an energy expenditure close to rest values, such as watching television, using the computer, playing video games, and other similar activities. The harm of SB on health has been shown to be somewhat independent of meeting current physical activity recommendations. The Brazil National Adolescent Health Promotion policy contains guidelines that encourage and promote physical activity but does not address SB and its repercussions [36].

Our results showed that sedentary behavior was also positively associated with regular alcohol or tobacco use in the last 30 days. The findings in the literature remain controversial. While similar results were reported in a study with adolescents (13–15 years old) living in Southeast Asia [37], where SB was positively associated with licit and illicit drug use, another study in Italy found a negative association of SB with drug consumption [38]. Sedentary behavior may be related to alcohol use because alcohol drinking is considered a normative behavior frequently associated with most social encounters [39].

Adolescents reporting unhealthy eating habits, i.e., fruit consumption <5 days/week and soft drink consumption ≥5 days/week, [17] had a higher SB prevalence. A sedentary lifestyle in adolescence appears to coexist with inappropriate eating habits [12, 24]. Habits acquired during adolescence may persist through adulthood, contributing to the occurrence of noncommunicable chronic diseases and eventual increased morbidity and mortality rates [3–7]. Time spent watching television might lead adolescents to a greater exposure of unhealthy food advertisements, favoring unhealthy food consumption preferences that can be worsened by parents’ dietary choices in an unhealthy familiar environment [32].

Regarding self-assessed health, SB was more prevalent among students who reported regular or poor/very poor health. Self-assessed health is more than just physical health, and includes overall well-being and psychosocial condition, which can be affected by various factors, such as sedentary lifestyle. Long periods of sitting time can impair an individual’s health perception by acting as a promoter of physical and mental illnesses [40].

## Limitations

PeNSE is a representative research providing data about the health status of Brazilian adolescents, and its results can be extrapolated and used for temporal comparison. However, as a cross-sectional study, it does not allow assumptions about the temporal and causal nature of observed associations. Additionally, regarding the SB definition, the prevalence may be underestimated because the SB question considered neither time spent at work nor at the school and the self-reported nature of the questionnaire. Finally, PeNSE 2015 did not provide anthropometric measures such as weight and height, which are often correlated to SB in the literature [8, 41]. Last, it was difficult to compare our findings with previous literature because of the adopted (narrow) SB definition, as well as the lack of consensus regarding a clinically-meaningful SB cut-off for adolescents.

## Conclusion

Understanding the factors associated with sedentary behavior is vital to enhance strategies that address health issues among sedentary adolescents and foster the development of appropriate public policies and interventions.

Although a cause-effect relationship cannot be established, we can observe that the presence of SB coexists with the presence of inadequate lifestyle habits, which may contribute to the emergence of short-term and long-term health-related problems in the study group. SB was also positively associated with non-modifiable variables, such as gender and age, which is fundamental to prioritize specific target groups for future intervention efforts.

Actions should be intersectoral and promoted in the adolescents’ context. The school environment, as well as the family context, should be environments conducive to health promotion. Our results can inform future public health policies, expanding current recommendations regarding physical activity or healthy eating to include SB-specific guidelines.

We highlight the need for more studies that investigate the SB using a broader definition, including different SB domains apart from screen time. We also claim for future research aimed at developing a scientifically and clinically meaningful SB cut-off point for adolescents. Further research assessing SB with objective measures among Brazilian adolescents must be encouraged.
